# IL-14α as a Putative Biomarker for Stratification of Dry Eye in Primary Sjögren’s Syndrome

**DOI:** 10.3389/fimmu.2021.673658

**Published:** 2021-05-03

**Authors:** Yichen Liang, Zhenhua Xian, Dehua Fu, Shuang Liu, Yang Yao, Yuebo Jin, Chun Gao, Long Shen, Guixiu Shi, Jing He

**Affiliations:** ^1^ Department of Rheumatology and Immunology, First Affiliated Hospital of Xiamen University, Xiamen, China; ^2^ Department of Gastrointestinal Surgery, Tongji Hospital of Tongji Medical College of Huazhong University of Science & Technology, Wuhan, China; ^3^ Department of Rheumatology and Immunology, Peking University People’s Hospital, Beijing, China; ^4^ Department of Oncology, Northern Jiangsu People’s Hospital, Yangzhou, China; ^5^ Department of Oncology, Cancer Institute Affiliated to Northern Jiangsu People’s Hospital, Yangzhou, China; ^6^ Clinical Medical College, Yangzhou University, Yangzhou, China

**Keywords:** interleukin 14 α, Taxilin, biomarker, dry eye, Sjögren’s syndrome

## Abstract

**Background:**

Dry eye is often the first presenting manifestation of primary Sjögren’s syndrome (pSS). Because of the high prevalence of dry eye disease in normal population, ophthalmologists urgently need a non-invasive and reliable screening test to diagnose dry eye associated SS patients, other than ocular symptoms and signs. Currently, there is no single test available. The correlation of serum IL-14α with pSS has been found in pSS mouse model.

**Purpose:**

To evaluate whether IL-14α can serve as a biomarker to stratify dry eye in primary Sjögren’s syndrome and its correlation to BAFF in a cohort of patients with non-SS dry eye (NSDE), pSS with dry eye disease, rheumatoid arthritis (RA), and healthy controls (HC).

**Methods:**

Retrospective study based on serum levels of IL-14α (defined by Western Blot) and BAFF (measured by ELISA) were evaluated among pSS with dry eye disease, NSDE, RA, and HC groups. Serum levels of SS related autoantibodies (Ro, La, SP1, PSP, and CA6) were also measured by ELISA.

**Results:**

One hundred and eighty patients were included for the current study, patients were separated into four groups as defined by pSS (n=65), NSDE (n=20), RA (n=50) and HC (n=45). The level of serum IL-14α in pSS was significantly higher compared to NSDE, RA, and HC (p=0.0011, p=0.0052 and p<0.0001, respectively). The levels of serum BAFF in pSS was significantly higher than in NSDE and HC (p=0.0148 and p<0.0001, respectively, whereas the levels of serum BAFF in RA was only significantly higher than in HC (p=0.001), but the level of BAFF was no significant difference between pSS and RA. In pSS, there was a decrease in the serum levels of IL-14α associated with a longer duration of the disease. Also, there was a correlation between the serum levels of IL-14α and SS related autoantibodies such as anti-SSA/Ro and anti-SSB/La in pSS patients.

**Conclusions:**

This is the first paper to report both IL-14α and BAFF could serve as a critical cytokine biomarker for the stratification of dry eye in primary Sjögren’s syndrome. This may help ophthalmologists to develop non-invasive metrics for the diagnosis of dry eye associated pSS.

## Highlights

- Interleukin 14, a B cell growth factor, plays an essential role in the early pathophysiology of pSS.
- IL-14α and BAFF may work in different pathways to maintain the abnormal B cell activation.
- Interleukin 14 can serve as a biomarker to stratify dry eye related to pSS from non-pSS dry eye.

## Introduction

Sjögren’s syndrome (SS) is a chronic autoimmune disease characterized by salivary and lacrimal gland destruction as well as the involvement of many other systemic organs (such as lung, kidney, and liver) (1–4). SS affects 0.5% of the population and shows a strong female predominance (1, 5). SS can be classified as either primary or secondary when it coexists with other systemic autoimmune diseases. Primary SS (pSS) is characterized by severe dry mouth (98% of pSS patients) and dry eye (93% of pSS patients) as the major symptoms due to inflammation and dysfunction of salivary and lacrimal glands (6, 7). About 4% of patients with pSS will develop non-Hodgkin lymphomas later on in their life (8–10). The estimated standard index ratio (SIR) of lymphomas complicating primary Sjögren’s syndrome is between 4.9 to 44.4 (11). Due to its diverse presentation of symptoms, there is a significant delay at an average time of 6.5 years in the diagnosis of SS (12). Early detection of pSS is critical for significantly improving the efficacy of biological treatment within the first 5 years of the therapeutic window of pSS (13).

Studies have shown that up to 10% of dry eye patients have SS, and it took approximately 12 years of the patients suffering from dry eye symptom before being diagnosed as pSS (14, 15).

Therefore, ophthalmologists are the first care provider to meet the patients with dry eye, who could play a key role to shorten the diagnostic time and effectively treat the patients in their early phase of pSS. However, conventionally, ocular symptoms and signs in isolation have been poorly predictive of extraocular objective signs required for the diagnosis of SS patients, as multiple autoimmune diseases may present with dry eye symptoms without any known symptoms specific for SS-related dry eye (16). A serological biomarker that helps stratify SS from the dry eye patients without SS would significantly improve the diagnosis of SS. Recent studies have shown IL-14α can induce pSS and may play an essential role in the development of related clinical symptoms including dry eye (17–19).

B cell hyperactivity is a dominant feature of SS, manifested by hypergammaglobulinemia, autoantibody production, and cryoglobulinemia (20). Most studies of B cell hyperactivity in SS focus on B cell activating factor (BAFF), a cytokine that promotes the survival and proliferation of B cells. Increased expression of BAFF is associated with the hyperactive B cell response in pSS (21, 22). BAFF expression level in pSS patient’s serum is enhanced and correlated with classic SS autoantibodies such as anti-SSA/Ro, anti-SSB/La, and rheumatoid factor (RF) (23). Another B cell growth factor, interleukin 14 (IL-14, also known as Taxilin), has also been shown to play an essential role in the pathophysiology of pSS (24). IL-14 can promote the proliferation of germinal center (GC) B cells (24). Previous studies have shown that IL-14 can selectively act on memory B cells to enhance its function (17, 25, 26). Based on recent reviews, it was further proposed that IL-14α can induce SS by converting low-affinity autoreactivity into high-affinity memory B cells (17, 26). Studies show that transgenic mice overexpressing human IL-14α (IL-14αTG) can develop many clinical features of pSS in the same relative time frame as seen in patients. In the IL14αTG mouse model, it showed a group of antibodies targeting specific antigens expressed in salivary and lacrimal gland tissue such as salivary gland protein 1 (SP1), carbonic anhydrase 6 (CA6), and parotid secretory protein (PSP), a group of autoantibodies, also known as tissue-specific autoantibodies (TSA) (17). The IL14αTG mouse also developed anti-SSA/Ro antibodies. However, the TSA developed were early in the SS disease course before the anti-SSA/Ro antibodies (17). These findings have also been found in human studies of SS and patients with idiopathic dry mouth and dry eye disease (27). Studies also suggest that the TSA antibodies may serve as serological biomarkers to identify early SS, particularly among patients who are seronegative for anti-SSA/Ro autoantibody (17–19, 27, 28).

In this study, we investigated the expression of IL-14α associated with dry eye patients of pSS. Our purpose is to evaluate whether IL-14α can serve as a biomarker to stratify dry eye and along with BAFF to correlate in a cohort of patients with non-SS dry eye (NSDE), pSS with dry eye disease, rheumatoid arthritis (RA), and healthy controls (HC).

## Material and Methods

### Study Group Design

Enrollment in the current study occurred between March 2016 and October 2017 in Peking University People^’^s Hospital. Institutional Review Board approval of the study protocol was obtained before the study. Informed consent was obtained from all subjects. Serum was collected and aliquoted before transferring to -80 °C freezer for long-term storage. To be enrolled in the study the subjects fulfilled the following criteria. The subjects in the pSS group had to meet the 2016 ACR/EULAR classification criteria for primary SS (29). The disease duration of pSS was determined by calculating the time elapse between disease onset point (through collecting the first manifestation of pSS and time of its emergence from patients’ medical records) and endpoint of this study (the last time to collect the patient serum). The RA patients had to meet ACR/EULAR 2010 rheumatoid arthritis classification criteria (30). The NSDE patients were first screened based on symptoms (dry eye questionnaire - DEQ-5 ≥ 6 or ocular surface disease index-OSDI ≥ 13) and then evaluated by homeostasis markers (Tear break up time (TBUT < 5S)) according to the 2017 dry eye workshop (DEWS) II Diagnostic Methodology report (31). NSDE patients were evaluated according to the 2016 ACR/EULAR classification criteria for primary SS. In general, NSDE patients has no SS related symptoms except dry eye. Further evaluation of autoantibodies such as ANA, the classical autoantibodies (Ro and La) and immunoglobulin levels were all negative for our NSDE patients. Tissue-specific autoantibodies (TSA), which had been reported as diagnostic biomarkers for pSS and appeared early in the disease, were also all negative for NSDE patients. The HC group subjects were selected based on the medical history. Only subjects without a history of any autoimmune disease were enrolled for this study. Furthermore, the NSDE and HC group also had to show the negative serology test results for SS related autoantibodies (SSA/SSB) to be included in the study.

### Western Blot

Western blot assays were run following manufacturer instructions as previously described (32). In brief, 4ul serum samples were taken from 1:100 diluted serum of patient, then the samples were mixed with 20ul PBS and 6ul 5X loading buffer. Mixed samples were boiled for 8 minutes and 15ul of the boiled mixed samples were loaded and separated by 10% SDS-PAGE gel and transferred onto a nitrocellulose membrane. The membrane was blocked for 2h at room temperature (RT) and incubated overnight at 4°C with 1:1000 IL-14α antibody (San Ying Biotechnology Co. Ltd., Wuhan, China) in a blocking buffer. After washing three times for 10 min each with TBST buffer, the membrane was incubated with the HRP conjugated goat anti-mouse IgG secondary antibody for 2h at RT. The membrane was washed three times for 10 min each with TBST before substrate was added to visualize the result using ChemiDoc™ gel imaging system (Bio-Rad, Hercules, CA).

### Calculation of the Relative Intensity Ratio for Serum IL-14α Levels

Since different batches of gels were run for multiple samples, after optimized the experimental condition, one negative IL-14α patient serum was chosen to be run in each western blot as internal control for the normalization of all the patient samples through the study, at the same time, one positive IL-14α patient serum was also used in each western blot as positive control for the reproducibility of results. A ratio of mean density reflecting the actual relative expression level of IL-14α was calculated (see **Data Sheet 4** and **Data Sheet 5** in **Supplementary Materials**).

### ELISA

To measure serum BAFF levels, ELISA assay was run following manufacturer instructions (R&D Systems, Minneapolis, MN) as previously described (32). For SS related autoantibodies (Ro, La, SP1, PSP, and CA6), ELISA kits were acquired from Trinity Biotech, Buffalo, NY, and run following manufacturer instructions. In brief, the plate was firstly washed three times with PBS containing 0.05% Tween-20 (PBST) before 100ul of the diluted samples and standards were added to separate well. After incubation for 2h at RT, the plate was washed three times with PBST again. 100uL per well of HRP labeled goat anti-human secondary antibody was added and incubated for 2h at RT. After washing three times with TBST, the plate was developed using TMB Peroxidase EIA substrate kit (Pierce, Rockford, IL, USA) and incubated for 10 min at RT. The reaction was stopped with 2M H_2_SO4, and the optical density was read at 450 nm on a Biotek ELISA reader (Biotek, Winooski, VT).

### Statistical Analysis

SPSS (version 16.0, IBM) or Prism (version 6.0, GraphPad Software) were used to analyze the statistical significance of data generated from the current study. Data were presented as mean ± standard deviation (SD). The unpaired two-tailed Student’s test was used to compare the difference between the two groups. Normal distribution of the data was assessed by Shapiro-Wilk test with P>0.05. Pearson correlation coefficient was used to analyze the correlations between two variables and P values < 0.05 were considered as statistically significant.

## Results

The patient population and their basic clinical characteristics were shown in **Table 1**. Patients were divided into four groups: 45 HC, 65 patients with pSS, 50 patients with RA, and 20 patients with NSDE. The HC were age and sex matched.

**Table 1:** Basic clinical characteristics of study groups.

Group	Healthy Control (HC)	primary Sjögren’s syndrome (pSS)	Rheumatoid Arthritis (RA)	non-SS dry eye (NSDE)	P value
Number of Cases	45	65	50	20	
Male/Female	18/27	2/63	17/23	0/20	
Age (years)*	43.27±14.78	53.15 ± 14.08	53.68 ± 15.26	44.85±11.39	
Ro (+/-)	0/45	58/7	0/50	0/20	
La (+/-)	0/45	30/35	0/50	0/20	
ANA (+/-)	0/45	61/4	13/37	0/20	
IgA (+/-)	0/45	12/53	5/45	0/20	
IgG (+/-)	0/45	37/28	7/43	0/20	
IgM (+/-)	0/45	10/55	3/47	0/20	
Ocular staining score*	NA	7.95±3.62	NA	4.25±2.15	<0.0001
Schirmer’s test* (mm)	NA	3.00±4.76	NA	10.4±8.29	0.0009
Tear film break-up time (s)*	NA	1.37±1.65	NA	3.25±0.64	<0.0001
OSDI*	NA	NA	NA	22.74±5.61	

*Values in **Table 1** were presented as Mean ± SD.

### The Mean Serum IL-14α and BAFF Levels in HC, NSDE, pSS, and RA Groups

**Figure 1:**
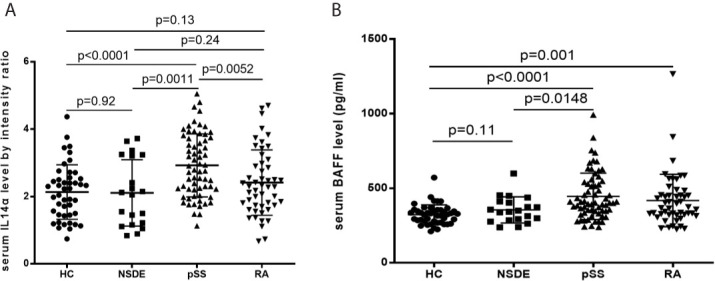
**(A)** Relative intensity ratio for serum IL-14α levels in HC, NSDE, pSS, and RA groups. **(B)** Serum BAFF levels in HC, NSDE, pSS, and RA groups.

The mean relative intensity ratio for serum IL-14α levels between the HC group was 2.13 ± 0.81, the NSDE group was 2.11 ± 0.99, the pSS group was 2.93 ± 0.93 and the RA group was 2.41 ± 0.97. Serum IL-14α levels in the pSS group significantly increased compared to the HC group (p<0.0001), the NSDE group (p=0.0011), and the RA group (p=0.0052), whereas serum IL-14α of RA had no significant difference compared that of HC or NSDE (p=0.13 and 0.24, respectively) (**Figure 1A**).

Mean serum BAFF levels (pg/ml) in the HC group were 323.56 ± 65.85, the NSDE group were 355.21 ± 87.86 (p=0.11), the pSS group were 455.94 ± 155.16 (p<0.0001) and the RA group were 418.15 ± 175.99 (p=0.001). The mean serum BAFF levels of the pSS group also showed a significant increase compared to that of the HC or NSDE group (p<0.0001 and p=0.0148, respectively). The mean serum BAFF level of the RA group also significantly increased compared to that of the HC group (p=0.001, respectively) (**Figure 1B**).

### The Association Between Serum IL-14α Levels and TSA Autoantibodies in Patients of pSS

**Figure 2:**
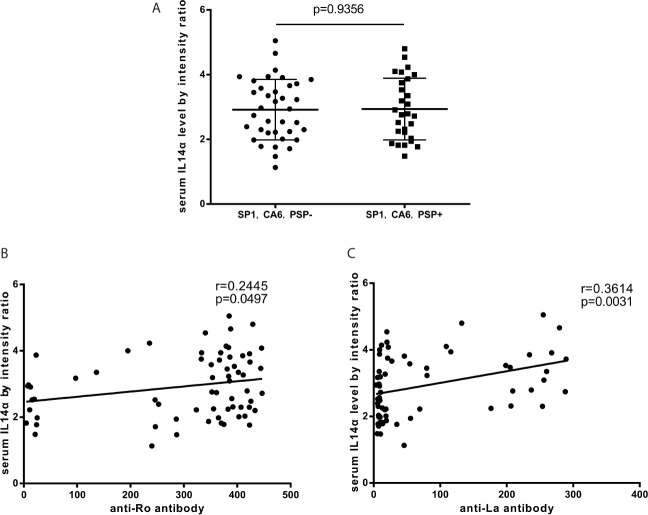
**(A)** The relationship between serum IL-14α levels and TSA panel (anti-SP1 antibody, anti-PSP antibody, and anti-CA6 antibody). **(B)** The relationship between serum IL-14α levels and anti-Ro antibody. **(C)** The relationship between serum IL-14α levels and anti-La antibody.

Tissue-specific autoantibodies (TSA) which include antibodies to salivary protein 1 (SP1), parotid secretory protein (PSP), and anti-carbonic anhydrase 6 (CA6) are reported to be diagnostic biomarkers of pSS and appeared early in the disease. The panel was deemed to be positive if any one of these three antibodies was positive. We found that there was no difference with serum IL-14α levels between TSA panel negative or panel positive patients (p=0.9356) (**Figure 2A**). The results showed that serum IL-14α levels significantly correlated with the classical autoantibodies anti-Ro (p=0.0497) (**Figure 2B**), and anti-La (p=0.0031) (**Figure 2C**).

### The Change of Serum IL-14α and BAFF Levels Associated With the Disease Duration of pSS

**Figure 3:**
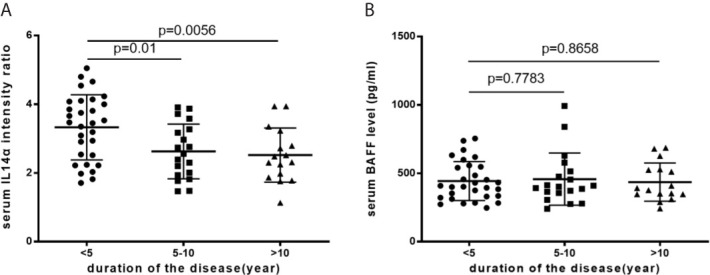
**(A)** The mean serum IL-14α levels with disease duration. **(B)** The mean serum BAFF levels with disease duration.

In pSS patients, the mean serum level of IL-14α was significantly higher in the disease duration less than 5 years compared to that of either longer than 5 years up to 10 years or longer than 10 years (3.33 ± 0.95 *vs* 2.63 ± 0.80, p=0.01; 3.33 ± 0.95 *vs* 2.52 ± 0.79, p=.0056; respectively) (**Figure 3A**). The serum levels of BAFF (pg/ml) was not change within different disease duration times [(<5 years, 443.82 ± 142.35; 5-10 years, 457.33 ± 191.09 (p=0.7783), and >10 years, 463.38 ± 139.55 (p=0.8658)] (**Figure 3B**).

## Discussion

Studies have shown that B cells play a central role in the pathogenesis of pSS. characterized by early polyclonal B-cell hyperactivity. There is a switch later in the disease process to the expansion of monoclonal B-cells that results in the development of B-cell lymphoma in pSS patients (8–11). The role of BAFF in the pathogenesis of pSS is well established (23). BAFF is a cytokine that promotes B cell maturation, proliferation, and survival, which has been well established in animal models (21–23). BAFF transgenic mice develop features of SLE and later clinical characteristics of pSS, such as sialadenitis. Approximately 3% of these mice develop lymphoma spontaneously between 12-18 months (33). Increased levels of BAFF in the salivary gland can induce B cell hyperactivity and contribute to SS pathogenesis (34, 35). The BAFF levels are associated with increased antigen production and disease activity scores in pSS patients (36, 37). Serum BAFF levels are enhanced and correlate with levels of classic SS related autoantibodies such as anti-Ro, anti-La, and RF in pSS (19).

IL-14, a B cell growth factor, has been shown to play an essential role in the pathophysiology of pSS. Studies have shown that the IL14αTG mice develop clinical symptoms of dryness of the mouth and eyes with foci of lymphocytic infiltration in the salivary glands and develop mucosal associated lymphomas as seen in the human pSS patients (17, 18, 26–28). The time frame of the IL14αTG developing symptoms mirrors the human pSS. The disease progression in the IL14αTG could be divided into four different stages. In the initial stage, there is minimal clinical manifestation but there are serological abnormalities of the development of TSA. No histological abnormalities in the salivary or lacrimal gland are noted. In the second stage, the clinical features of dry mouth and/or eye become evident. There is also mild to moderate lymphocytic infiltration of lacrimal and submandibular glands. Stage three of the disease is characterized by systemic organ involvement. Moderate lymphocytic infiltration is seen in the salivary, lacrimal glands, lungs, kidney, pancreas, and liver. Stage four shows moderate to severe lymphocytic infiltration of all affected organs. B cell mucosal associated lymphoma is observed, mainly in the gut. Based on pSS progression in this animal model, TSA is identified and later found in patients with SS both together and without classic anti-Ro autoantibody, as well as in patients with idiopathic dry mouth and dry eye disease (27).

While both IL14 and BAFF transgenic mice share lots of similar features an animal model for pSS, such as lymphocytic infiltration of the lacrimal and submandibular gland (38, 39), there are significant differences between these two animal models. For example, BAFF transgenic mice do not spontaneously develop lymphoma, as in IL14α TG mice. BAFF transgenic mice also develop more severe proliferative glomerulonephritis compared to IL14αTG mice (40). The correlation of BAFF with pSS has been well established in multiple clinical studies (20, 23). Previous studies of IL-14α in pSS focused on animal models, and human studies are scarce. In the only clinical study involving the human subject, IL-14α gene expression is shown to be overexpressed in the peripheral blood leukocytes (18). Data from the current study provide strong evidence to support that IL-14, like BAFF, is a critical B cell related cytokine and can promote autoantibody production in SS pathogenesis. Serum IL-14α levels in pSS correlated well with the classical autoantibodies anti-Ro, and anti-La. Serum IL-14α and BAFF levels were significantly increased in pSS group compared to HC and NSDE group. Both IL-14α and BAFF could serve as a critical cytokine biomarker for the stratification of pSS from NSDE.

While the difference between RA and pSS group was not significant for serum BAFF levels, there was a significant difference between RA and pSS group for serum IL-14α levels. In pSS patients, the serum levels of IL-14α were higher within the first 5 years of disease duration, whereas the serum levels of BAFF did not. Based on these observations, we speculate that IL-14α and BAFF may work in different ways to maintain the abnormal B cell activation as seen in pSS patients. The correlation of these cytokines and autoantibodies may provide new insights to understand the early disease progression in the pathogenesis of pSS and hence may help to find novel therapeutic targets for the treatment of pSS.

Future follow-up longitudinal studies of recently diagnosed dry eye patients with pSS and their cytokines levels, mainly IL-14α and BAFF, have been planned, to elucidate any correlation.

## Data Availability Statement

The raw data supporting the conclusions of this article will be made available by the authors, without undue reservation.

## Ethics Statement

The studies involving human participants were reviewed and approved by Institutional Review Board, Peking University People’s Hospital. The patients/participants provided their written informed consent to participate in this study.

## Author Contributions

JH, GS, and LS contributed to conception and design of the study. YL, ZX, DF, SL, YY, YJ, and CG helped with the acquisition of data. YL performed the statistical analysis. YL wrote the first draft of the manuscript. ZX, DF, SL, and LS wrote sections of the manuscript. All authors contributed to the article and approved the submitted version.

## Funding

This research was partly supported by National Science funding of China (Grant No. U1605223) and National Program on Key Basic Research Project (973 Program, No. 2014CB541903) to GS.

## Conflict of Interest

The authors declare that the research was conducted in the absence of any commercial or financial relationships that could be construed as a potential conflict of interest.
