# Supplementary material for: IL-14α as a Putative Biomarker for Stratification of Dry Eye in Primary Sjögren’s Syndrome
Source: Front Immunol. 2021 May 3;12:673658. doi: 10.3389/fimmu.2021.673658 (PMC8126710; doi:10.3389/fimmu.2021.673658)
Supplement: Supplementary file 1 [file DataSheet_1.docx]

| 2016 American College of Rheumatology/European League Against Rheumatism classification criteria for primary Sjögren’s syndrome: The classification of primary Sjögren’s syndrome (SS) applies to any individual who meets the inclusion criteria,* does not have any of the conditions listed as exclusion criteria,† and has a score of ≥4 when the weights from the five criteria items below are summed | |
| --- | --- |
|  | Score |
| Labial salivary gland with focal lymphocytic sialadenitis and focus score of ≥1 foci/4 mm2 ‡ | 3 |
| Anti-SSA/Ro-positive | 3 |
| Ocular Staining Score ≥5 (or van Bijsterveld 1 score ≥4) in at least one eye §¶ | 1 |
| Schirmer’s test ≤5 mm/5 min in at least one eye § | 1 |
| Unstimulated whole saliva flow rate ≤0.1 mL/min§** | 1 |

*These inclusion criteria are applicable to any patient with at least one symptom of ocular or oral dryness, defined as a positive response to at least one of the following questions: (1) Have you had daily, persistent, troublesome dry eyes for more than
3 months? (2) Do you have a recurrent sensation of sand or gravel in the eyes? (3) Do you use tear substitutes more than three times a day? (4) Have you had a daily feeling of dry mouth for more than 3 months? (5) Do you frequently drink liquids to aid in swallowing dry food? or in whom there is suspicion of Sjögren’s syndrome (SS) from the European League Against Rheumatism SS Disease Activity Index questionnaire (at least one domain with a positive item).

†Exclusion criteria include prior diagnosis of any of the following conditions, which would exclude diagnosis of SS and participation in SS studies or therapeutic trials because of overlapping clinical features or interference with criteria tests: (1) history of head and neck radiation treatment, (2) active hepatitis C infection (with confirmation by PCR), (3) AIDS, (4) sarcoidosis, (5) amyloidosis, (6) graft-versus-host disease, (7) IgG4-related disease.

‡The histopathologic examination should be performed by a pathologist with expertise in the diagnosis of focal lymphocytic sialadenitis and focus score count, using the protocol described by Daniels et al.

§Patients who are normally taking anticholinergic drugs should be evaluated for objective signs of salivary hypofunction and ocular dryness after a sufficient interval without these medications in order for these components to be a valid measure of oral and ocular dryness.

¶Ocular Staining Score described by Whitcher et al; van Bijsterveld score described by van Bijsterveld.

**Unstimulated whole saliva flow rate measurement described by Navazesh and Kumar.
