# Supplementary material for: IL-14α as a Putative Biomarker for Stratification of Dry Eye in Primary Sjögren’s Syndrome
Source: Front Immunol. 2021 May 3;12:673658. doi: 10.3389/fimmu.2021.673658 (PMC8126710; doi:10.3389/fimmu.2021.673658)
Supplement: Supplementary file 2 [file DataSheet_2.docx]

The 2010 American College of Rheumatology/European League Against Rheumatism classification criteria for rheumatoid arthritis

| score | |
| --- | --- |
| Target population (Who should be tested?): Patients who |  |
| 1) have at least 1 joint with definite clinical synovitis (swelling)* |  |
| 2) with the synovitis not better explained by another disease† |  |
| Classification criteria for RA (score-based algorithm: add score of categories A–D; a score of ≥6/10 is needed for classification of a patient as having definite RA) ‡ |  |
| A. Joint involvement§ |  |
| 1 large joint ¶ | 0 |
| 2-10 large joints | 1 |
| 1-3 small joints (with or without involvement of large joints) # | 2 |
| 4-10 small joints (with or without involvement of large joints) | 3 |
| >10 joints (at least 1 small joint) ** | 5 |
| B. Serology (at least 1 test result is needed for classification) †† |  |
| Negative RF *and* negative ACPA | 0 |
| Low-positive RF *or* low-positive ACPA | 2 |
| High-positive RF *or* high-positive ACPA | 3 |
| C. Acute-phase reactants (at least 1 test result is needed for classification) ‡‡ |  |
| Normal CRP *and* normal ESR | 0 |
| Abnormal CRP *or* abnormal ESR | 1 |
| D. Duration of symptoms §§ |  |
| <6 weeks | 0 |
| ≥6weeks | 1 |

* The criteria are aimed at classification of newly presenting patients. In addition, patients with erosive disease typical of rheumatoid arthritis (RA) with a history compatible with prior fulfillment of the 2010 criteria should be classified as having RA. Patients with longstanding disease, including those whose disease is inactive (with or without treatment) who, based on retrospectively available data, have previously fulfilled the 2010 criteria should be classified as having RA.

† Differential diagnoses vary among patients with different presentations, but may include conditions such as systemic lupus erythematosus, psoriatic arthritis, and gout. If it is unclear about the relevant differential diagnoses to consider, an expert rheumatologist should be consulted.

‡ Although patients with a score of <6/10 are not classifiable as having RA, their status can be reassessed and the criteria might be fulfilled cumulatively over time.

§ Joint involvement refers to any *swollen* or *tender* joint on examination, which may be confirmed by imaging evidence of synovitis. Distal interphalangeal joints, first carpometacarpal joints, and first metatarsophalangeal joints are *excluded from assessment*. Categories of joint distribution are classified according to the location and number of involved joints, with placement into the highest category possible based on the pattern of joint involvement.

¶ “Large joints” refers to shoulders, elbows, hips, knees, and ankles.

# “Small joints” refers to the metacarpophalangeal joints, proximal interphalangeal joints, second through fifth metatarsophalangeal joints, thumb interphalangeal joints, and wrists.

** In this category, at least 1 of the involved joints must be a small joint; the other joints can include any combination of large and additional small joints, as well as other joints not specifically listed elsewhere (e.g., temporomandibular, acromioclavicular, sternoclavicular, etc.).

†† Negative refers to IU values that are less than or equal to the upper limit of normal (ULN) for the laboratory and assay; low-positive refers to IU values that are higher than the ULN but ≤3 times the ULN for the laboratory and assay; high-positive refers to IU values that are >3 times the ULN for the laboratory and assay. Where rheumatoid factor (RF) information is only available as positive or negative, a positive result should be scored as low-positive for RF. ACPA = anti-citrullinated protein antibody.

‡‡ Normal/abnormal is determined by local laboratory standards. CRP = C-reactive protein; ESR = erythrocyte sedimentation rate.

§§ Duration of symptoms refers to patient self-report of the duration of signs or symptoms of synovitis (e.g., pain, swelling, tenderness) of joints that are clinically involved at the time of assessment, regardless of treatment status.
