# Supplementary material for: IL-14α as a Putative Biomarker for Stratification of Dry Eye in Primary Sjögren’s Syndrome
Source: Front Immunol. 2021 May 3;12:673658. doi: 10.3389/fimmu.2021.673658 (PMC8126710; doi:10.3389/fimmu.2021.673658)
Supplement: Supplementary file 4 [file DataSheet_4.pdf]

The following information is an updated version of a method for using ImageJ to analyze western blots from a now-deprecated older page. Don't use the alternate methods discussed on the old page, as they are subject to way too much user bias.

ImageJ (http://rsb.info.nih.gov/ij/index.html) can be used to compare the density (aka intensity) of bands on an agar gel or western blot. This tutorial assumes that you have carried your gel or blot through the visualization step, so that you have a digital image of your gel in .tif, .jpg, .png or other image formats (.tif would be the preferred format to retain the maximum amount of information in the original image). If you are scanning x-ray film on a flatbed scanner, make sure you use a scanner with the ability to scan transparencies (i.e. film). See the references at the end of this tutorial for a discussion of the various ways that you can screw this step up.

The method outlined here uses the Gel Analysis method outlined in the ImageJ documentation: http://rsb.info.nih.gov/ij/docs/menus/analyze.html#gels. You may prefer to use it instead of the methods I outline below. There should be very little difference between the results obtained from the various methods. This version of the tutorial was created using ImageJ 1.42q on a Windows 7 64-bit install.

1. Open the image file using *File>Open* in ImageJ.
2. The gel analysis routine requires the image to be a gray-scale image. The simplest method to convert to grayscale is to go to *Image>Type>8-bit*. Your image should look like Figure 1.

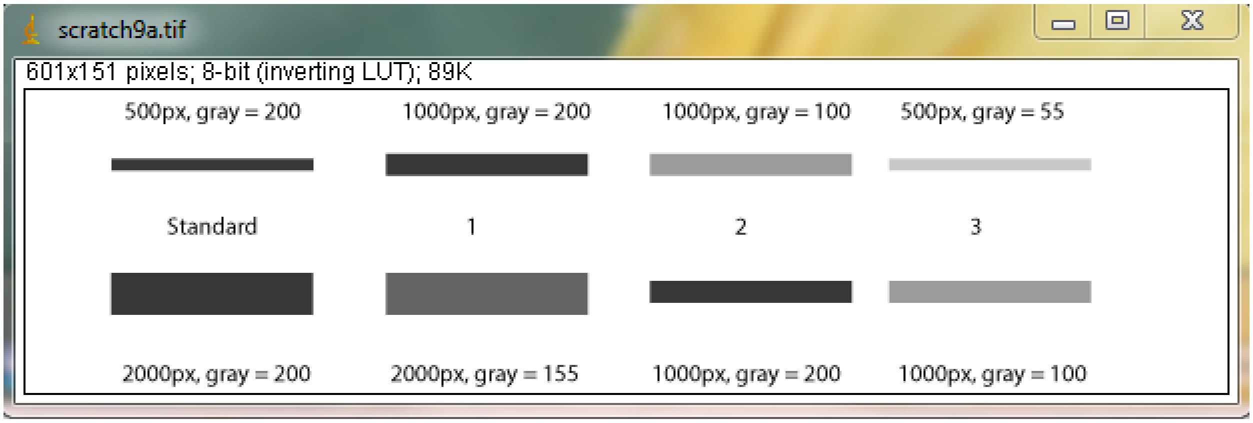

Figure 1. A fabricated western blot image opened in ImageJ. The information along the top of the image indicates that the image is currently in 8-bit mode, using an inverting LUT (look-up table). The inverting LUT ensures that dark bands will be recorded as higher density values.

3. Choose the **Rectangular Selections** tool from the ImageJ toolbar. Draw a rectangle around the first lane. ImageJ assumes that your lanes run vertically (so individual bands are horizontal), so your rectangle should be tall and narrow to enclose a single lane. If you draw a rectangle that is short and wide, ImageJ will switch to assuming the lanes run horizontally (individual bands are vertical), leading to much confusion.

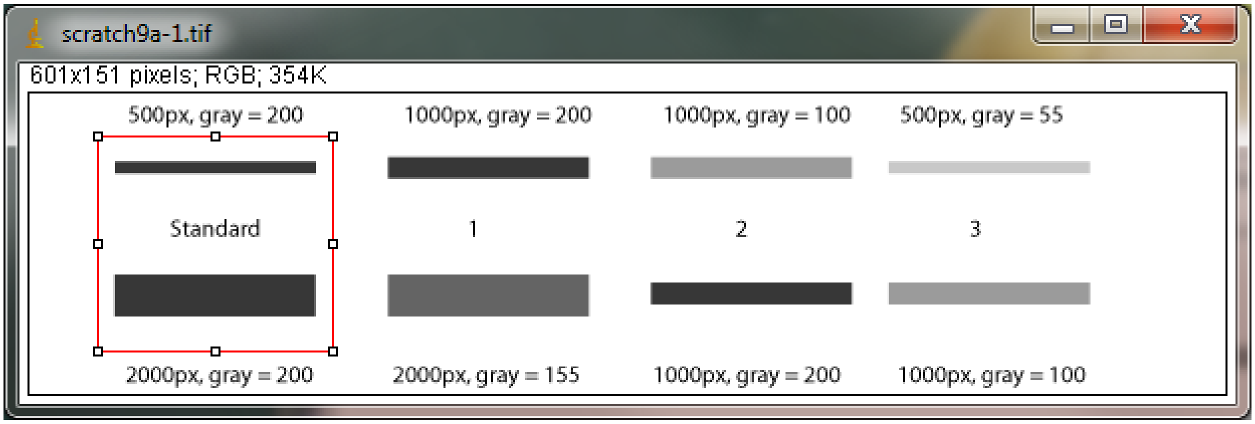

Figure 2. The first lane has been selected with the Rectangular Selections tool

4. After drawing the rectangle over your first lane, press the **1** key or go to *Analyze>Gels>Select First Lane* to set the rectangle in place. The 1st lane will now be highlighted and have a 1 in the middle of it.
5. Use your mouse to click and hold in the middle of the rectangle on the 1st lane and drag it over to the next lane. You can also use the arrow keys to move the rectangle, though this is slower. Center the rectangle over the lane left-to-right, but don't worry about lining it up perfectly on the same vertical axis. ImageJ will automatically align the rectangle on the same vertical axis as the 1st rectangle in the next step.
6. Press **2** or go to *Analyze>Gels>Select Next Lane* to set the rectangle in place over the 2nd lane. A 2 will appear in the lane when the rectangle is placed.
7. Repeat Steps 5 + 6 for each subsequent lane on the gel, pressing **2** each time to set the rectangle in place (Figure 3).

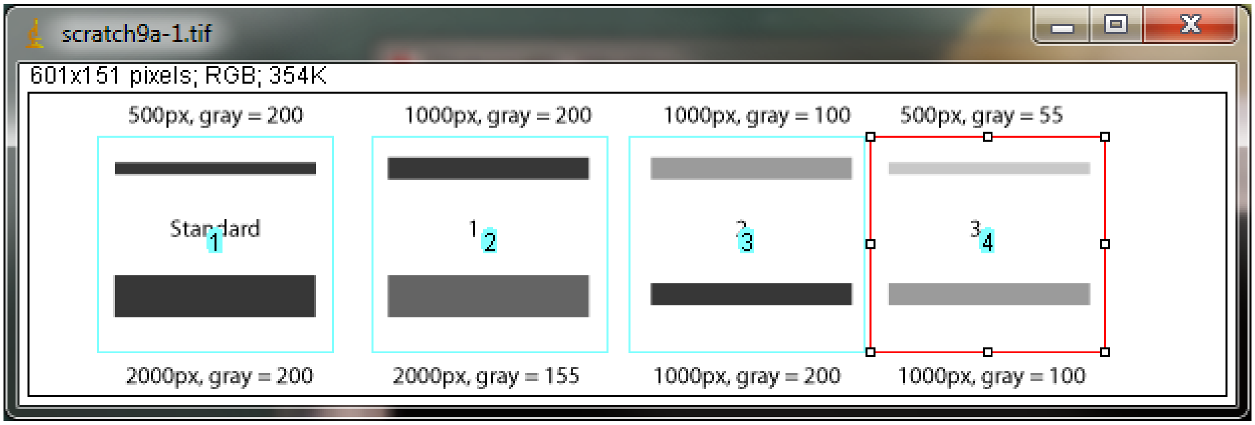

Figure 3. Place the rectangle over each lane, pressing 2 each time to set the rectangle in place.

8. After you have set the rectangle in place on the last lane (by pressing **2**), press **3**, or go to *Analyze>Gels>Plot Lanes* to draw a profile plot of each lane.

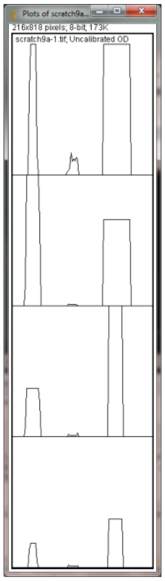

Figure 4. The profile plot from the example western blot.

9. The profile plot represents the relative density of the contents of the rectangle over each lane. The rectangles are arranged top to bottom on the profile plot. In the example western blot image, the peaks in the profile plot (Figure 4) correspond to the dark bands in the original image (Figure 3). Because there were four lanes selected, there are four sections in the profile plot. Higher peaks represent darker bands. Wider peaks represent bands that cover a wider size range on the original gel.
10. Images of real gels or western blots will always have some background signal, so the peaks don't reach down to the baseline of the profile plot. Figure 5 shows a peak from a real blot where there was some background noise, so the peak appears to float above the baseline of the profile plot. It will be necessary to close off the peak so that we can measure its size.

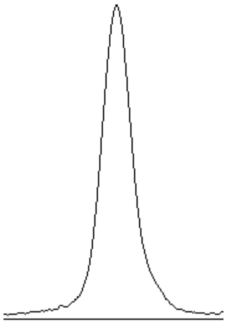

Figure 5. Real western blots often have some background noise, so the peak doesn't reach down to the baseline (= perfect white).

11. Choose the **Straight Line** selection tool from the ImageJ toolbar (Figure 6). For each peak you want to analyze in the profile plot, draw a line across the base of the peak to enclose the peak (Figure 5). This step requires some subjective judgment on your part to decide where the peak ends and the background noise begins.

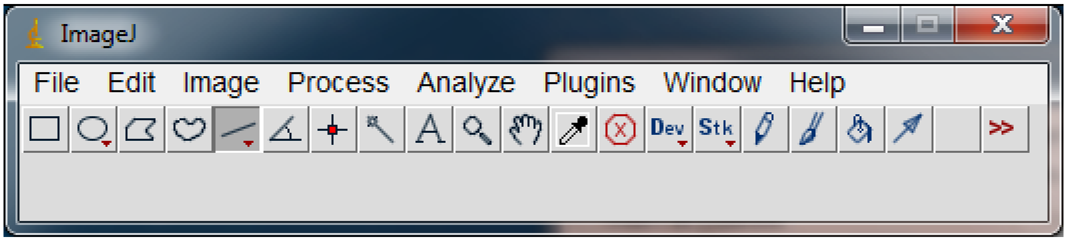

Figure 6. Use the straight line tool to close off the base of each peak of interest.

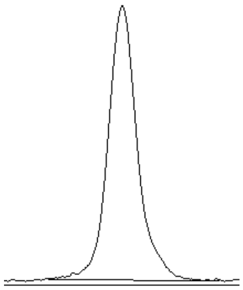

Figure 7. The peak from Figure 5 is shown with a line drawn across the base of the peak to enclose the area of the peak.

12. Note that if you have many lanes highlighted, the later lanes will be hidden at the bottom of the profile plot window. To see these lanes, press and hold the space bar, and use the mouse to click and drag the profile plot upwards.
13. When each peak has been closed off at the base with the **Straight Line** selection tool, select the **Wand** tool from the ImageJ toolbar (Figure 8).

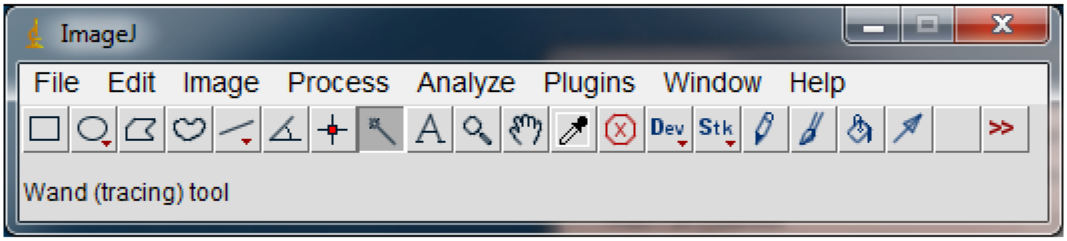

Figure 8. Choose the Wand tool to highlight each peak of interest on the profile plot.

14. Using the spacebar and mouse, drag the profile plot back down until you are back at the first lane. With the **Wand** tool, click inside the peak (Figure 9). Repeat this for each peak as you go down the profile plot. For each peak that you highlight, measurements should pop up in the Results window that appears.

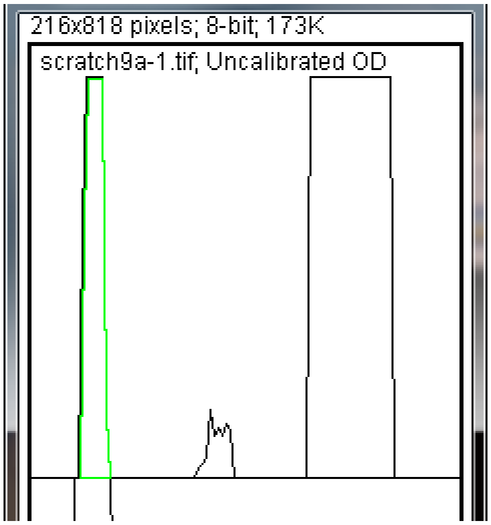

Figure 9. Click inside the 1st peak of interest with the Wand tool. The peak should be highlighted and a measurement should pop up in the Results window.

15. When all of the peaks have been highlighted, go to *Analyze>Gels>Label Peaks*. This labels each peak with its size, expressed as a percentage of the total size of all of the highlighted peaks.
16. The values from the Results window (Figure 10) can be moved to a spreadsheet program by selecting *Edit>Copy All* in the Results window. Paste the values into a spreadsheet.

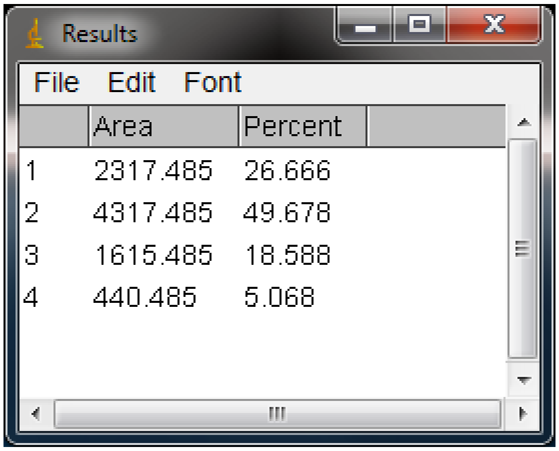

	Area	Percent
1	2317.485	26.666
2	4317.485	49.678
3	1615.485	18.588
4	440.485	5.068

Figure 10. The output from selecting peaks in the profile plot and labeling the peaks. In this case, the results are from selecting the upper band in each of the 4 lanes in the example western blot from Figure 1.

Note: If you accidentally click in the wrong place with the **Wand**, the program still records that clicked area as a peak, and it will factor into the total area used to calculate the percentage values. Obviously this will skew your results if you click in areas that aren't peaks. If you do

happen to click in the wrong place, simple go to *Analyze>Gel>Label Peaks* to plot the current results, which displays the incorrect values, but more importantly resets the counter for the Results window. Go back to the profile plot and begin clicking inside the peaks again, starting with the 1st peak of interest. The Results window should clear and begin showing your new values. When you're sure you've click in all of the correct peaks without accidentally clicking in any wrong areas, you can go back to *Analyze>Gels>Label Peaks* and get the correct results.

Data analysis

With your data pasted into a spreadsheet, you can now calculate the relative density of the peaks. As a reminder, the values calculated by ImageJ are essentially arbitrary numbers, they only have meaning within the context of the set of peaks that you selected on the single gel image you've been working on. They do not have units of µg of protein or any other real-world units that you can think of. The normal procedure is to express the density of the selected bands relative to some standard band that you also selected during this process.

1. Place your data in a spreadsheet. One of the peaks should be your standard. In this example we'll use the 1st peak as the standard.
2. In a new column next to the Percent column, divide the Percent value for each sample by the Percent value for the standard (the 1st peak in this case, 26.666).
3. The resulting column of values is a measure of the relative density of each peak, compared to the standard, which will obviously have a relative density of 1.

Sample	Area	Percent	Relative Density
1	2317.485	26.666	1.00
2	4317.485	49.678	1.86
3	1615.485	18.588	0.70
4	440.485	5.068	0.19

Figure 11. Calculating relative density for each of the 4 highlighted peaks. We use Sample 1 as the standard for this gel. The Relative Density is calculated by dividing the Percent value for each sample by the Percent value for the standard.

4. In this example, the 2nd lane has a higher Relative Density (1.86), which corresponds well with the size and darkness of that band in the original image (Figure 1). Recall that these data are for the upper row of bands on the original western blot image.
5. If you want to compare the density of samples on multiple gels or blots, you will need to use the same standard sample on every gel to provide a common reference when you calculate Relative Density values. See the sections below for more detailed discussion of these requirements.
6. In order to test for significant differences between treatments in an experiment, all of your gels or blots will need to be scanned and quantified using this method, and the values will be expressed in terms of Relative Density, or you can treat Relative Density

as a fold-change value (i.e. a Relative Density difference of 2 between a control and treatment would indicate a 2-fold change in expression). If you will be using analysis of variance techniques to test your data, you may need to ensure that your Relative Density values are normally distributed and that there is homogeneity of variance among the different treatments.

7. It should be noted here that some researchers make the extra effort to include a set of serial dilutions of a known standard on each blot. Using the serial dilution curve and the quantification techniques outlined above, it should be possible to express your sample bands in terms of picograms or nanograms of protein

A more involved example using loading-controls.

We'll use Figure 12 as a representative western blot. On this blot, we will pretend that we loaded four replicate samples of protein (four pipette loads out of the same vial of homogenate), so we expect the densities in each lane to be equivalent. The upper row of bars will represent our protein of interest. The lower set of bars will represent our loading-control protein, which is meant to ensure that an equal amount of *total* protein was loaded in each lane. This loading-control protein is a protein that is presumably expressed at a constant level regardless of the treatment applied to the original organisms, such as actin (though many people will question the assertion that actin will be expressed equivalently across treatments).

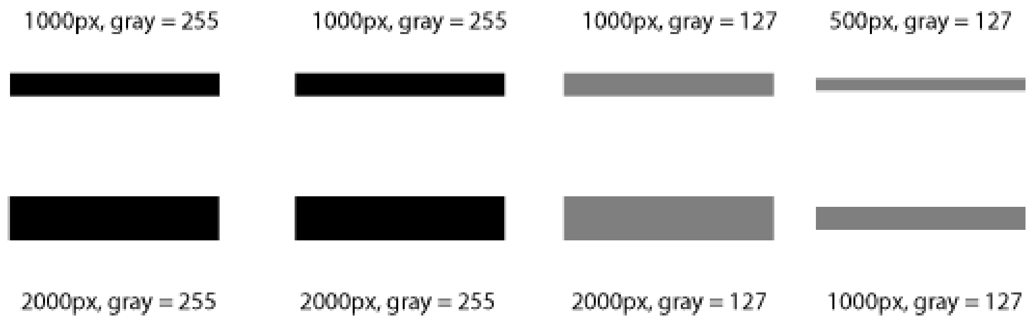

Figure 12. An example western blot with a lower row of loading-control bands. Often these bands will represent a protein that is thought to be expressed equivalently in all treatments, such as actin.

Looking at Figure 12, we had hoped to load equivalent amounts of total protein in each lane, but after running the western blot, the size and intensity of the lower bars in each lane varies quite a lot. The two left lanes appear equivalent, but the 3rd lane has half the density (gray value) compared to lanes 1+2, while lane 4 has half the density and half the size compared to lanes 1+2. Because our loading controls are so different, the density values of the upper set of bands may not be directly comparable.

We'll use ImageJ's gel analysis routine to quantify the density and size of the blots, and use the results from our loading-controls (lower bands) to scale the values for our protein of interest (upper bands).

1. Open the western blot image in ImageJ.
2. Make sure that the image is in 8-bit mode: go to *Image>Type>8-bit*.
3. Use the rectangle tool to draw a box around the entire 1st lane (both upper and lower bars included.
4. Press "1" to set the rectangle. A "1" should appear in the middle of the rectangle.
5. Click and hold in the middle of the rectangle and drag it over the 2nd lane.
6. Press "2" to set the rectangle for lane 2. A "2" should appear in the middle of the rectangle.
7. Repeat steps 5 + 6 for each subsequent lane, pressing "2" to set the rectangle over each subsequent lane (see Figure 13).

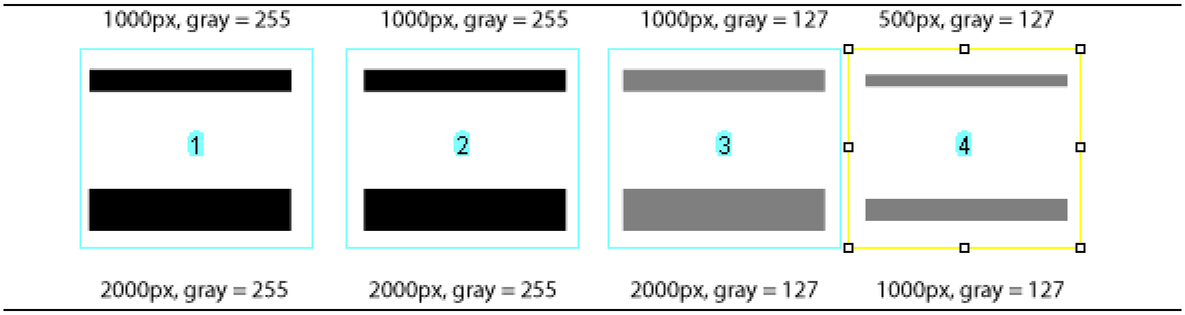

Figure 13. Each lane has been selected by moving the rectangle over it and pressing "2" to set the rectangle in place.

8. When you have placed the last lane (and pressed "2" to set it in place), you can press "3" to produce a plot of the selected lanes (see Figure 14).

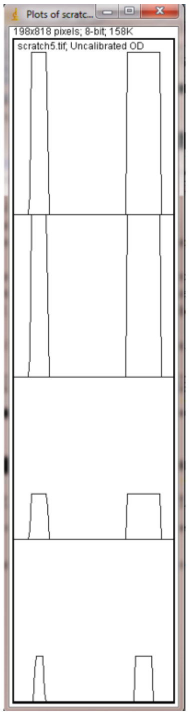

Figure 14. Profile plot of each lane (arranged with lane 1 at the top, lane 4 at the bottom). You may need to scroll through the window to see all of the lanes.

9. The profile plot essentially represents the average density value across a set of horizontal slices of each lane. Darker blots will have higher peaks, and blots that cover a larger size range (kD) will have wider peaks. In our example western blot, the bands are perfect rectangles, but you will notice some slope in the profile plot peaks, as ImageJ is applying a bit of averaging of density values as it moves from top to bottom of each lane. As a result, the sharp transition from perfect white to perfect black on the bands of lane 1 is translated into a slight slope on the profile plot due to the averaging.
10. On our idealized western blot used here, there is no background noise, so the peak reaches all the way down to the baseline of the profile plot. In real western blots, there will be some background noise (the background will not be perfectly white), so the peaks won't reach the baseline of the profile plot (Figure 15). As a result, each plot will need to have a line drawn across the base of the peak to close it off.

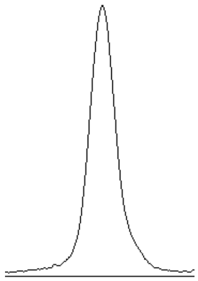

Figure 15. Real western blots often have some background noise, so the peak doesn't reach down to the baseline (= perfect white).

11. Choose the **Straight Line** selection tool from the ImageJ toolbar. For each peak you want to analyze in the profile plot, draw a line across the base of the peak to enclose the peak (Figure 16). This step requires some subjective judgment on your part to decide where the peak ends and the background noise begins.

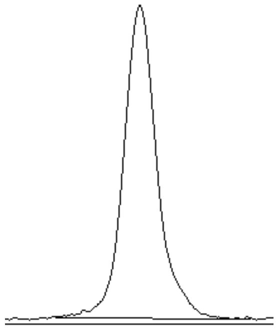

Figure 16. The peak from Figure 15 is shown with a line drawn across the base of the peak to enclose the area of the peak.

12. When each peak of interest is closed off with the straight line tool, switch to the **Wand** tool. We will use the wand tool to highlight each peak of interest so that ImageJ can calculate its relative area+density.
13. We will start by highlighting the loading-control bands (lower row) on our example western blot. Beginning at the top of the profile plot, use the wand to click inside the 1st peak (Figure 17). The peak should be highlighted after you click on it. Continue clicking on the loading-control peaks for the other lanes. If a lane is not visible at the bottom of the profile plot, hold down the space bar and click-and-drag the profile plot upwards to reveal the remaining lanes.

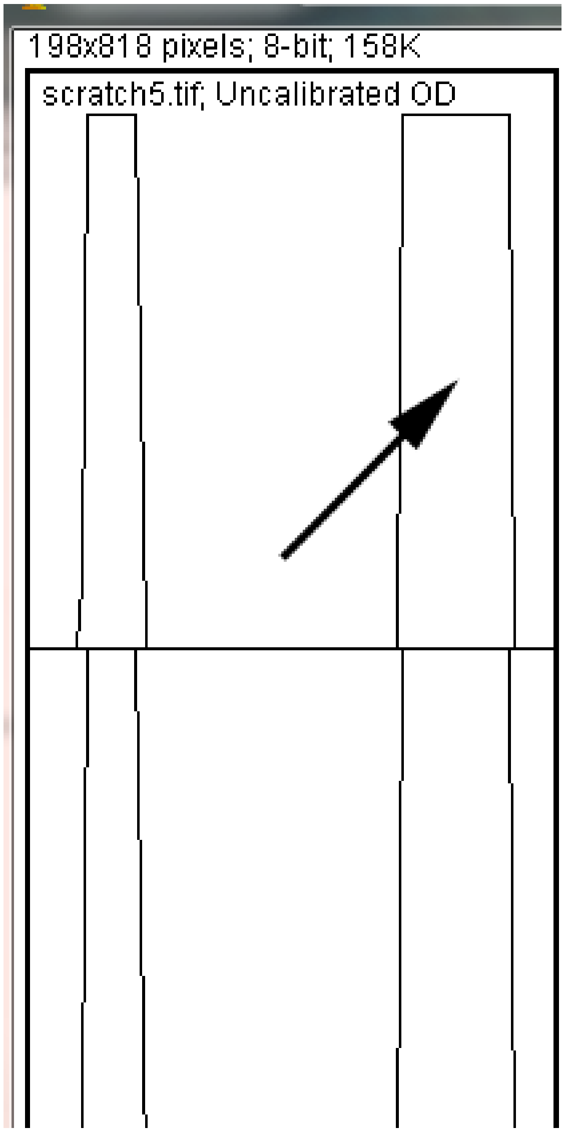

Figure 17. Click inside the loading-control peak with the wand tool. Click on each of the loading-control peaks for the remaining lanes (ignore the protein lanes to the left for now).

14. When the loading control peak for each lane has been highlighted with the wand, go to *Analyze>Gel>Label Peaks*. Each highlighted peak will be labeled with its relative size expressed as a percentage of the total area of all the highlighted peaks. You can go to the Results window and choose *Edit>Copy All* to copy the results for placement in a spreadsheet.

stds	Area	Percent
1	8398.657	40.828
2	8398.657	40.828
3	2476.657	12.04
4	1296.657	6.303

Figure 18. Area and Percent values for our loading-control bands from the example western blot. Lanes 1 and 2 are equal, as we expect by looking at the original bands on the western blot (Figure 12).

15. Repeat steps 13 + 14 for the real sample peaks now. We are selecting these peaks separately from the loading-control peaks so that those areas are not factored into the calculation of the density of our proteins-of-interest. As before, use the **Wand** tool to click inside the area of the peak in the 1st lane, then continue clicking inside the peaks of the remaining lanes. When finished, go to *Analyze>Gel>Label Peaks* to show the results. Copy the results to a spreadsheet alongside the data for the loading-control bands (Figure 19).

stds	Area	Percent
1	8398.657	40.828
2	8398.657	40.828
3	2476.657	12.04
4	1296.657	6.303
Samples	Area	Percent
1	4265.485	40.585
2	4265.485	40.585
3	1284.485	12.222
4	694.485	6.608

Figure 19. Results from the loading-control bands (labeled "stds" here) and the sample protein peaks (the upper row of bands on the original western blot).

Data Analysis

1. With all of the relative density values now in the spreadsheet, we can calculate the relative amounts of protein on the western blot. Remember that the "Area" and "Percent" values returned by ImageJ are expressed as relative values, based only on the peaks that you highlighted on the gel. Start the analysis by calculating Relative Density values for each of the loading-standard bands. In this case, we'll pretend that Lane 1 is our control that we want to compare the other 3 lanes to. Divide the Percent value for each lane by the Percent value in the control (Lane 1 here) to get a set of density values that is relative to the amount of protein in Lane 1's loading-control band (Figure 20).

stds	Area	Percent	Rel. Density
1	8398.657	40.828	1
2	8398.657	40.828	1
3	2476.657	12.04	0.294896
4	1296.657	6.303	0.154379

Figure 20. Calculate Relative Density values by dividing the Percent values in each row by the control sample's Percent value (Lane 1, 40.828, in our example).

2. Next we'll calculate the Relative Density values for our sample protein bands (upper row on the example western blot). We carry out a similar calculation as step 1, dividing the Percent value in each row by the Percent value of our control's protein band (Lane 1 here).

stds	Area	Percent	Rel. Density
1	8398.657	40.828	1
2	8398.657	40.828	1
3	2476.657	12.04	0.294896
4	1296.657	6.303	0.154379
Samples	Area	Percent	Rel. Density
1	4265.485	40.585	1
2	4265.485	40.585	1
3	1284.485	12.222	0.301146
4	694.485	6.608	0.162819

Figure 21. Calculate Relative Density values for the Samples as well, using the Percent value for your control protein band (Lane 1, 40.585 here).

Note: Recall that because some of our loading-control bands were wildly different on the original western blot, we can't simply use the Relative Density values from our Samples calculated in Step 2 as the final results. Now it is necessary to scale the Relative Density values for the Samples by the Relative Density of the corresponding loading-control bands for each lane. We do this based on the assumption that the proportional differences in the Relative Densities of the loading-control bands represent the proportional differences in amounts of total protein we loaded on the gel. In our example western blot, we have evidence of massively different amounts of total protein in each sample (poor pipetting practice, probably).

3. The final step is to scale our Sample Relative Densities using the Relative Densities of the loading-controls. On the spreadsheet, divide the Sample Relative Density of each lane by the loading-control Relative Density for that same lane.

stds	Area	Percent	Rel. Density	
1	8398.657	40.828	1	
2	8398.657	40.828	1	
3	2476.657	12.04	0.294896	
4	1296.657	6.303	0.154379	
Samples	Area	Percent	Rel. Densi	Adj. Density
1	4265.485	40.585	1	1
2	4265.485	40.585	1	1
3	1284.485	12.222	0.301146	1.021194
4	694.485	6.608	0.162819	1.054667

Figure 22. Adjusted Density values (yellow cells) for our samples were calculated by dividing the Relative Density of each Sample lane by the Relative Density of the loading-control for the same lane. For example, the Relative Density for Sample 4 (0.1628) was divided by the Relative Density for the loading-control in Lane 4 (0.154379) to get an Adjusted Density value of 1.054.

4. When we created our imaginary example western blot, we expected to get equivalent amounts of the protein of interest in each lane, because we (hypothetically) loaded equivalent sample volumes of protein from the same vial of homogenate in each lane. During the loading process, something went awry, and we got very different densities on the western blot. But after carrying out the loading-control corrections above, we find that our Adjusted Densities for each of the 4 lanes are roughly equivalent (all are approximately = 1), indicating that there are equivalent ratios of the protein of interest contained within the sample of total protein in each lane, as we would expect.

The example above represents a special case. In reality, you will typically be skilled enough to pipette equivalent amounts of total protein into your gel lanes, presumably because you have used a process like the BCA Protein Assay to quantify the total amount of protein present in each of your homogenate samples. This can obviate the need for the loading-control bands and the associated controversy about whether the protein you're using to assess equal loading is truly expressed consistently across all treatment levels.

Extending the example to multiple western blots

If you can apply equal amounts of total protein to each lane of a gel, then you only need to concern yourself with having an appropriate standard sample placed on each western blot you run for the project. For example, you might purchase an aliquot of purified Human Hsp70 and place 1µl in a lane on each gel you run when probing for Hsp70. Or you might save some money by creating your own standard sample by mixing aliquots of several of your homogenates to make a large quantity of mixed homogenate that you can use to load a standard volume onto a single lane of each gel for your entire project (running out of this mixture part way through your

project would be a Bad Thing). In this case you may not know the true concentration of the protein of interest in your mixed homogenate, but you will at least be able to get an equivalent amount of total protein (including the protein of interest) on each gel.

The goal of the standard sample is to account for variations in antibody binding efficiency (for example if you're re-using a mixture of antibody on multiple western blots to save money), for variations in blocking and washing efficiency, for variation in the decay rate of a chemiluminescent reagent used to visualize the antibodies, or for variation in the exposure time of your x-ray film. In any of these cases, the intensity of the signal you get out of your western blot can vary from blot to blot (Figure 23). You can use a standard sample on each gel to normalize every other band on that same blot, and then compare across multiple blots because every band in your dataset is normalized to the same standard (be it 10ng of Human Hsp70 or a mixture of several homogenates).

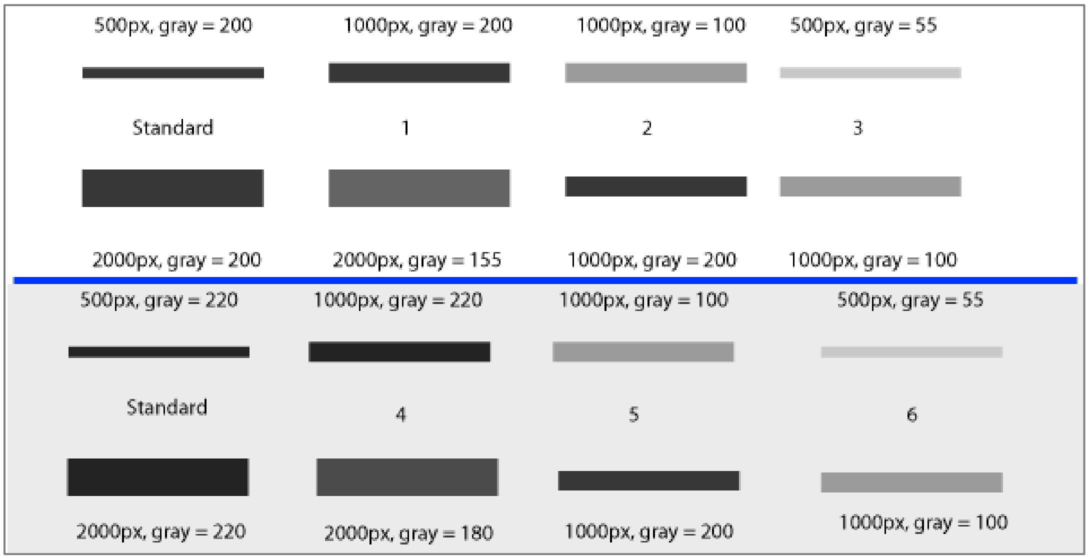

Figure 23. Two example western blots with a common standard sample (Lane 1). The binding/incubation/development steps of these two blots varied slightly, resulting in a different final exposure which alters the absolute densities on the two blots. Because we have a standard sample on both blots, which is an identical volume of an identical protein sample (i.e. drawn from a common aliquot of homogenate), we can express the densities of the other lanes on each gel relative to the standard sample, eliminating the effects of the differing exposures.

Finally, if you are using a loading-control protein band (lower row on these blots) on each gel, and a standard protein sample on multiple gels, you can correct both for differences in total protein loaded in a lane (using the loading-control band) and for differences in exposure between gels (using the standard sample).

For example, consider the two gels in Figure 24. The 1st (white) gel has minimal background noise, and there is some variation the amount of protein loaded in each lane, as revealed by the size and density of the loading control bands of the samples. The 1st lane on the left is our standard sample used on each gel. The 2nd (gray) gel has more background noise due to a

different exposure time, poor washing, or any number of possible problems. Again, there are different protein quantities loaded in the 4 lanes, based on the size and density of the loading-control bands on the lower half. The 1st lane on the left is the same standard protein sample as that used on the 1st gel.

We would start by analyzing the two gels separately, using the protocol at the beginning of this document.

1. Calculate the relative density of the loading-control bands (lower row on this gel). Use the loading-control band for the standard in lane 1 to do this calculation.
2. Calculate the relative density of the bands for the protein band of interest (upper row on this gel). Use the protein band of interest for the standard in lane 1 to do this calculation.
3. Adjust the relative densities calculated in Step 2 using the relative densities for the loading-control bands calculated in Step 1. Simply divide the relative density from Step 2 for each lane by the corresponding relative density from Step 1.

After you've scaled the density of the protein of interest for each sample to the standard sample, and adjusted the relative density based on the loading-control band, you are left with an estimate of the relative density of the protein of interest in each lane on your gel (relative to the standard in lane 1). Repeat this for each separate gel. When finished, you'll note that because every sample lane is normalized to the standard sample in Lane 1 on that particular gel, and every gel contains the same standard sample, your cross-gel comparison is already accomplished, since every sample is now normalized to the same standard sample (Figure 24).

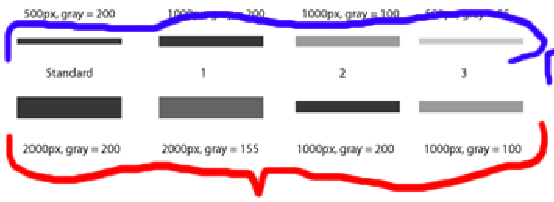

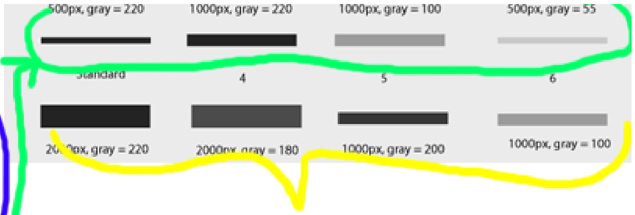

Load-control band	Area	Percent	Rel. Density
Standard	8398.657	42.335	1
1	5458.657	27.515	0.649935
2	4398.657	22.172	0.523727
3	1582.657	7.978	0.188449

Sample band	Area	Percent	Rel. Density	Adj. Density
Standard	2311.485	26.809	1	1
1	4309.485	49.983	1.864411	2.868612
2	1571.485	18.227	0.679884	1.298163
3	429.485	4.981	0.185796	0.98592

Load-control band	Area	Percent	Rel. Density
Standard	8398.657	45.041	1
4	5542.657	29.725	0.659954
5	3562.657	19.106	0.424191
6	1142.657	6.128	0.136054

Sample band	Area	Percent	Rel. Density	Adj. Density
Standard	2297.485	28.812	1	1
4	4301.485	53.944	1.872275	2.836978
5	1135.485	14.24	0.494239	1.165131
6	239.485	3.003	0.104227	0.766075

Figure 24. Example data from two western blots. The Relative Densities of the loading-control bands (lower bands on each gel, red and yellow lines) was calculated, using the value for the standard in Lane 1 (lower band) on each gel. The Relative Densities for the sample bands (upper row on each gel, blue and green lines) were calculated separately, and were normalized relative to the standard in Lane 1 (upper band). The "Adjusted Density" for each sample lane was calculated by dividing the sample relative density by the loading-control relative density for each lane separately (i.e. for sample 3, 0.185796/0.188449 = 0.98592).

After all of this work, you're left with (adjusted) Relative Densities for each of your samples (the yellow cells in Figure 24). At this point you can ignore the values of the standard samples, since you only needed those to ensure that your cross-gel comparisons were scaled properly. Now you can finally begin calculating average relative density values for your experimental control samples (maybe these are samples 3+6) and your experimental treatment samples (perhaps these are samples 1+4 and 2+5) to carry out the real comparison of protein expression under your treatment conditions. Because your relative density values are all normalized to the standard sample, you may wish to eventually re-scale your relative density values once again using your experimental controls' average value as a baseline.

A few extra details:

With regard to the ImageJ gel analysis routine, there has been some question of what the values reported by ImageJ correspond to. The images below may help illustrate what ImageJ is measuring.

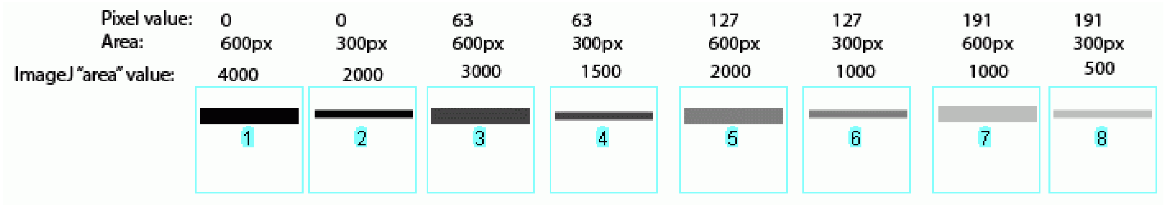

Figure 25. A comparison of "area" values returned by ImageJ.

In Figure 25 above, I have drawn out a set of fake "bands" in Adobe Illustrator. The gray value and area of each band are listed above the band. Additionally, I have included the "area" value returned by ImageJ after plotting the bands and clicking in each peak with the Wand tool. Note that these "area" values are a RELATIVE measure of the size and density of each peak you clicked with the wand tool. When you halve the area of a band, but maintain the same gray value (compare lanes 1+2), the value reported by ImageJ is half as large. By the same token, if you halve the gray value but maintain the same area (compare lanes 1+5), the value reported by ImageJ is halved.

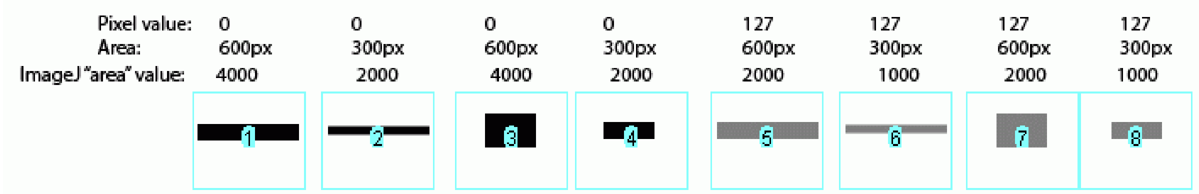

Figure 26. ImageJ will return similar area values when bands have equivalent numbers of pixels of equal darkness, but vary in shape.

The same holds true for bands of different shapes. In Figure 26 above, altering the shape of the band, but maintaining the same gray value and area (compare lanes 1+3) yields an equivalent value from ImageJ.

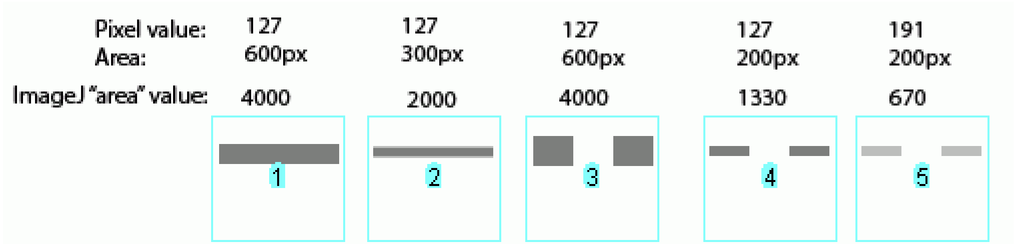

Figure 27. ImageJ deals with gaps in bands just fine. Note however that the "area" values returned by ImageJ are different compared to Figure 26 and Figure 27, because the number of lanes, size of the bands, and density of the bands has changed.

ImageJ also accounts for gaps in a band, as shown in Figure 27 above. Compare lanes 1+3, which both have an equal number of gray pixels and equal gray values (i.e. equal amounts of protein on the gel). ImageJ reports the same "area" value for both of these lanes.
It is worth reiterating that the "area" values and percentages reported by ImageJ are always relative to the total size and density of bands that you have selected in a particular image. In the image immediately above, the band in column 1 returns an "area" value of 4000, while in the previous two images column 1 had the same size band, but with twice the gray value, which in both cases also returned a value of 4000. The raw values returned by ImageJ are meaningless for comparing across different gels, since they are only a relative measure of the bands you've highlighted on a particular gel image. This is why we need to standardize to some common standard loaded onto all of the gels.

Additional references - The methods outlined here are by no means canonical and there are plenty of subtleties to be considered in this sort of analysis. For more practical considerations of scanning and analyzing gel images, see the following papers:

Gassmann, M., Grenacher, B., Rohde, B. and Vogel, J. (2009). Quantifying western blots: pitfalls of densitometry. *Electrophoresis* **30**, 1845-1855. doi: 10.1002/elps.200800720

Tan, H. Y. and Ng, T. W. (2008). Accurate step wedge calibration for densitometry of electrophoresis gels. *Optics Communications* **281**, 3013-3017. doi: 10.1016/j.optcom.2008.01.012
