# Supplementary material for: IL-14α as a Putative Biomarker for Stratification of Dry Eye in Primary Sjögren’s Syndrome
Source: Front Immunol. 2021 May 3;12:673658. doi: 10.3389/fimmu.2021.673658 (PMC8126710; doi:10.3389/fimmu.2021.673658)
Supplement: Supplementary file 5 [file DataSheet_5.docx]

Gel 1

**
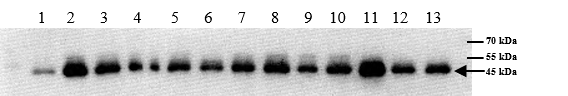
**

| Lane | Sample ID | Mean density value | Relative IL14α value |
| --- | --- | --- | --- |
| 1 | Internal control | 42.99 | 1.00 |
| 2 | Positive control | 176.36 | 4.10 |
| 3 | RA | 150.85 | 3.51 |
| 4 | QC fail / void | 102.15 | 2.38 |
| 5 | SS | 125.05 | 2.91 |
| 6 | SS | 110.11 | 2.56 |
| 7 | RA | 139.32 | 3.24 |
| 8 | SS | 161.32 | 3.75 |
| 9 | RA | 107.49 | 2.50 |
| 10 | NSDE | 153.98 | 3.58 |
| 11 | SS | 200.49 | 4.66 |
| 12 | HC | 128.14 | 2.98 |
| 13 | HC | 132.22 | 3.08 |

Gel 2

**
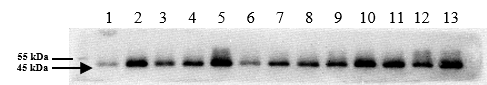
**

| Lane | Sample ID | Mean density vale | Relative IL14α value |
| --- | --- | --- | --- |
| 1 | Internal control | 40.13 | 1.00 |
| 2 | Positive control | 160.66 | 4.00 |
| 3 | SS | 101.82 | 2.54 |
| 4 | SS | 129.44 | 3.23 |
| 5 | SS | 182.24 | 4.54 |
| 6 | NSDE | 69.79 | 1.74 |
| 7 | NSDE | 118.54 | 2.95 |
| 8 | RA | 124.32 | 3.10 |
| 9 | RA | 137.46 | 3.43 |
| 10 | RA | 178.89 | 4.46 |
| 11 | RA | 169.79 | 4.23 |
| 12 | RA | 143.79 | 3.58 |
| 13 | HC | 175.4 | 4.37 |

Gel 3


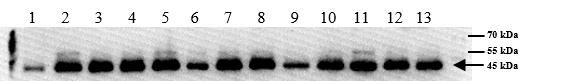


| Lane | Sample ID | Mean density vale | Relative IL14α value |
| --- | --- | --- | --- |
| 1 | Internal control | 47.48 | 1.00 |
| 2 | Positive control | 176.42 | 3.72 |
| 3 | NSDE | 172.72 | 3.64 |
| 4 | SS | 182.95 | 3.85 |
| 5 | SS | 187.09 | 3.94 |
| 6 | SS | 117.30 | 2.47 |
| 7 | SS | 183.80 | 3.87 |
| 8 | SS | 182.97 | 3.85 |
| 9 | RA | 101.20 | 2.13 |
| 10 | RA | 165.82 | 3.49 |
| 11 | RA | 177.43 | 3.74 |
| 12 | RA | 163.72 | 3.45 |
| 13 | HC | 157.15 | 3.31 |
