# Supplementary figures and images for: IL-14α as a Putative Biomarker for Stratification of Dry Eye in Primary Sjögren’s Syndrome
Source: Front Immunol. 2021 May 3;12:673658. doi: 10.3389/fimmu.2021.673658 (PMC8126710; doi:10.3389/fimmu.2021.673658)

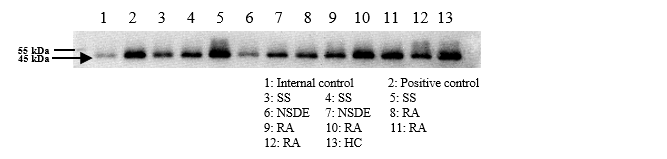

Supplement: Supplementary file 6 [file Image_1.tif]

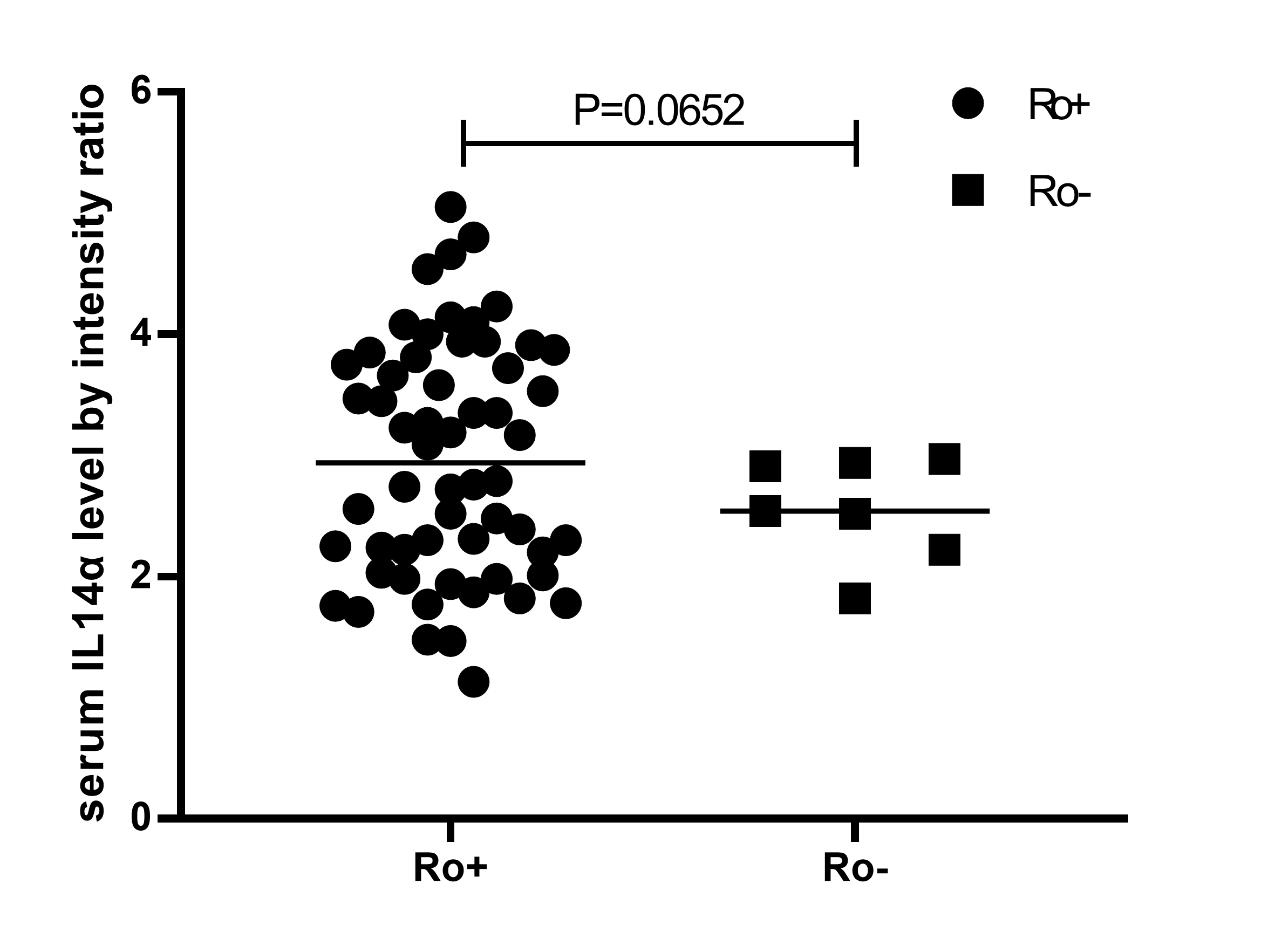

Supplement: Supplementary file 7 [file Image_2.tif]
